# Prospective and Retrospective Time Estimates of Children: A Comparison Based on Ecological Tasks

**DOI:** 10.1371/journal.pone.0033049

**Published:** 2012-03-06

**Authors:** Nicolas Bisson, Simon Tobin, Simon Grondin

**Affiliations:** École de psychologie, Université Laval, Québec, Québec, Canada; Duke University, United States of America

## Abstract

Children's time estimation literature lacks of studies comparing prospective and retrospective time estimates of long lasting ecological tasks, i.e. tasks reflecting children's daily activities. In the present study, children were asked to estimate prospectively or retrospectively how much time they played a video game or read a magazine. Regardless of the task, the results revealed that prospective time estimates were longer than the retrospective ones. Also, time estimates of the video game task were longer, less accurate and more variable than those of the reading task. The results are discussed in the light of the current literature about time estimation of long lasting ecological tasks.

## Introduction

From an adaptive stand point, time estimation is an important ability that individuals need to master in order to adapt to their environment. On that regard, literature on time estimation draws a distinction between prospective and retrospective timing [1], [2], [3], [4], [5]. In the former case, participants are informed in advance that they will have to judge time, while, in the latter case, they are told they will have to do so only after they have completed a task. In both situations, time judgments are made after the task is over. However, while they execute the task, participants of the prospective condition are aware that a time judgment will be required, while participants from the retrospective group are uninformed of this additional request. Since time estimation is made at the same moment, i.e. once the task is over, the key difference between these two conditions is that in the prospective paradigm, participants are aware that time is a critical component during the task, and therefore, can allow more attentional resources to time [6]. Thus, whereas prospective timing is reported to depend mainly on the amount of attention dedicated to time —with more attention to time resulting in longer perceived duration— retrospective timing is based mostly on memory processes and the number along with complexity of events that occur during the period to be timed, with more events and higher complexity resulting in longer perceived duration [3], [7]. Generally speaking, prospective time estimates are reported to be longer and less variable than retrospective time estimates [3]. Although researchers have been interested in understanding the paradigm differences with adult participants, very few have studied them with children samples.

Literature on children's prospective time estimation indicates that they become more sensitive to time between 3- and 8-years old [8], [9], [10]. Indeed, some authors report that children are less sensitive at 3- than at 5-years old, and that both groups are less sensitive than children at 8 [11], [12]. Moreover, some authors have reported that 8-year old children are prone to the same temporal illusions or effects than adults are: empty intervals are overproduced compared to filled intervals [13] and detracting attention from time results in temporal underestimation [10], [14]. Even if these findings are relevant for a better understanding of the developmental trajectory for time estimation by humans, some limitations could be addressed to this portion of the timing and time perception literature. First of all, there is a lack of studies comparing directly both time estimation paradigms (i.e. prospective vs. retrospective) with children samples. Secondly, there is a lack of studies addressing the capabilities of children for estimating long intervals (i.e. in the range of minutes), intervals marked by a task having some ecological validity. These two issues will be further discussed below.

As underlined by many authors, there is a need in the time estimation literature for studies where prospective and retrospective paradigms would be compared within the same task [3], [15]. This observation also applies specifically to the time estimation literature emphasizing the participation of children. As a matter of fact, of all the studies involving children participants that can be found in this literature, very few have used the retrospective paradigm. Moreover, in a recent meta-analysis on the effect of the cognitive load on prospective and retrospective time estimates, none of the data which contributed to the reported effect sizes came from studies comparing both paradigms in a children sample [1]. Finally, as Block, Zakay and Hancock [16] mentioned, comparing children's, adolescent's and adult's retrospective time estimates could provide the literature with an important theoretical and practical developmental knowledge about the processes involved in time estimation. As cognitive processes implied in time estimation (e.g. attention, memory) develop as children advance in age, a first step toward developmental comparisons could be to assess both time estimation paradigms in a children sample. This approach could then provide the occasion to verify if the results replicate what is generally found in the adolescent's and adult's time estimation literatures.

Another weakness of the psychological time literature that also applies to children research concerns the nature of the tasks used in the studies. Indeed, as pointed out by Tobin et al. [15], and almost five decades ago by Orme [17], this literature does not offer much about the perception of time involving ecological tasks. Such tasks can be defined as long lasting tasks reflecting daily activities like reading or playing a video game. On the contrary, time perception researches normally use non ecological tasks (e.g. pure tones marking the onset and offset of a 500-ms empty interval) in order to enhance the control on the experimental situation.

As a matter of fact, time estimation studies are usually interested in duration ranging from 100 ms to few seconds [5]. Children time estimation literature is not an exception. Indeed, in his literature review of children's time estimation, Friedman [18] mentions that most studies involving children used short duration intervals, normally 10 seconds or less. Moreover, out of the 20 studies included in Block and Zakay's [3] meta-analytic review concerning paradigms' comparison, only three used durations over 4 minutes: from 7.75 to 13.9 minutes [19], from 7.7 to 19.6 minutes [20] and 60 minutes [21]. Since this 1997 meta-analysis, only two studies concerned with a direct comparison of both paradigms and long durations were published: 8 or 24 minutes [22] and 12, 35 or 58 minutes [15]. Furthermore, of all studies that used durations above four minutes, only Tobin and Grondin [22] used a non-adult sample (participants were 14 or 15 years old).

Besides the duration issue, the nature of the task used in children's time estimation literature can be judged as quite different from what children are asked to do on a daily basis. For instance, the filled-duration effect was tested with children. This effect reveals that filled intervals are generally perceived as longer than empty intervals of the same length. To explore the filled-duration effect with children, researchers normally use a variety of stimuli to fill the temporal intervals: tones [11], [13], [23], [24], geometrical form pictures [10], [23]–[27], drawings [10], [24] and light bulbs [14]. Yet, in daily activities, children are asked to estimate durations of more complicated stimuli (e.g. book reading or video game play periods) than those used in the previously cited studies about the filled-duration effect.

In brief, these critics highlight the need to compare both time estimation paradigms with more ecological tasks, i.e. daily activities lasting more than 10 seconds. Indeed, tasks that are used in most experiments do not reflect the temporal demands of day to day tasks in children's life. Thus, the conclusions drawn from “non-ecological” tasks may not apply to other daily situations in which time perception is involved. With that in mind, the present study was designed to fill this gap in the literature.

The main objective of this study is to compare children's prospective and retrospective time estimates when they execute an ecological task: playing a video game. The reason behind the choice of children as participants is related to the fact that no study has ever compared prospective and retrospective time estimates with this population. Moreover, video gaming as an ecological task is a relevant choice when one considers that video gaming is a normal activity in most of children's daily life. Indeed, it takes up a large amount of children and adolescents' leisure time: up to 16 hours a week for boys and up to 9 for girls [28]. Therefore, selecting such an activity in the context of a time estimation study would be most relevant if the purpose is to estimate children's timing capabilities in a real-life situation (an ecologically valid task).

As stated earlier, only one known study has used a non-adult sample to compare prospective and retrospective time estimates with an ecologically valid task, i.e., with a task different from those normally used. Indeed, Tobin and Grondin [22] tested the hypothesis that adolescents play video games for long periods of time because they underestimate their play time. Thus, they asked adolescents of 14 and 15 years old to prospectively or retrospectively estimate the duration of three consecutive tasks: play a computer video game called Tetris (8 or 24 minutes), read a text about Einstein on a computer screen (8 minutes) and finally, play the video game (8 or 24 minutes). Half the participants were asked to prospectively estimate the duration of the three tasks while the other half had to estimate them retrospectively. Contrary to what is normally reported in time estimation literature, Tobin and Grondin [22] reported no paradigm effect: prospective and retrospective time estimates were not significantly different. Moreover, results showed that time estimates from both paradigms behaved like retrospective time estimates normally do, that is being shorter than the real duration. The authors argued that the absence of a difference between prospective and retrospective time estimates might have been caused by the fact that participants in both paradigm conditions were asked to estimate the duration of three tasks at once. Consequently, this manipulation might have changed the prospective nature of time estimates. Indeed, when the prospective paradigm is used in a study, durations are normally estimated one at the time, not three at the same time after durations have been presented to the participant. Also of interest is the study of Tobin et al. [15], which used a young adults sample (mean age of participants was 22.4 years old). Indeed, the authors have compared prospective and retrospective time estimates of long duration (12, 35 and 42 minutes) video game play periods in a computer video game center. In brief, the authors reported that prospective time estimates were: (a) longer, (b) not more or less accurate and (c) not more or less variable compared to retrospective time estimates. Therefore, we predict that, regardless of the task, children's prospective time estimates will be: (a) significantly longer (i.e. overestimated), (b) not significantly more or less accurate and (c) not significantly more or less variable than the retrospective ones.

Also, as gamers report loosing track of play time [29], it is important to consider the fact that time estimates of video game play periods can be different than those of other tasks. Thus, video game time estimates will be compared to those of a pleasant reading task. In their study with adolescents, Tobin and Grondin [22] reported that the duration of the video game period was more underestimated than the duration of a reading period. To explain these results, Tobin and Grondin argued that, in comparison with the reading task, the video game used required more cognitive resources (e.g. attention). Consequently, fewer resources were available during the video game condition to process temporal information, resulting in shorter time estimates. Thus, in the present study, we predict that children's time estimates of the video game task will be significantly underestimated compared to the reading task ones.

## Method

### Participants

This study included 199 participants (94 boys and 105 girls), aged from 8 to 12 years old (*M* = 9.42, *SD* = 1.11). Participants were taken in four different elementary schools. To keep the socio-economical level constant across experimental groups, all schools selected were located in the same part of the Québec City region. No *a priori* exclusion criteria were applied.

### Material

The video game used in this study is called “The Sims: Deluxe edition”. In this game, players can create their own micro-society and take charge of the characters' vital and social needs. Two basic scenarios were prepared in advance for participants: a household with a father and son, and a household with a mother and daughter. The game and the two basic scenarios were installed on multiple Windows XP stations in the computer laboratory of each school.

For the reading task, we used a French magazine for children called *Astrapi*. The magazine included mainly articles and educational comics. There was more than enough reading material for the 14-minute activity.

Two questionnaires were completed by participants on an Excel file. In the first one, participants were asked to indicate the number of minutes, and seconds, that corresponded to the length of either their playtime or reading period. They were then asked to indicate the likely minimal and maximal duration of their activity.

The second one was developed in-house and focused on socio-demographic data (age, gender, educational level, etc.). Finally, as the participant's level of enjoyment and feeling of competency might differ across conditions (e.g. task, duration and paradigm) or could be related to the three dependent variables, participants were asked to report both variables on two different Likert scales. At first, they were asked to report their task appreciation level (“On this 1 to 7 scale, how did you find this game”) on a 7 points Likert scale (1 = “Really boring” and 7 = “Really fun”). After, they reported their feeling of competency level (“On this 1 to 4 scale, how would you describe your competency level, i.e. your impression of having been good at this game”) on a 4 points Likert scale (1 = “Bad” and 4 = “Really good”).

### Procedure

This experiment was approved by Laval University's research ethical committee (*Comités d'éthique de la recherche avec des êtres humains de l'Université Laval*). Here is the procedure used to manage informed consent, as the ethical committee approved it. First, the researchers briefed the principals and the teachers of the selected elementary schools about the experiment (i.e. objectives, duration and experimental procedure) and their verbal consent was necessary to start recruiting the participants. Of relevance, a teacher could refuse to participate in the study, even if his principal or other teachers of their school had agreed to participate. Secondly, once a teacher accepted to participate in the study, the researchers planned the experimental schedule with the teacher so the study would not interfere with their educational program. Finally, in order to participate, all children had to return a consent form signed by their parents. Those who did not get their parents' approval were not eligible to participate in the study.

Participants were taken to the school computer lab during a normal class period, after having been asked to leave any personal belongings in class. In addition, classes were randomly assigned to one of the four following conditions: video game – prospective, video game – retrospective, reading – prospective and reading – retrospective. Descriptive statistics associated with these four conditions can be seen in Table 1 (gender statistics) and Table 2 (age statistics). Groups assigned to retrospective timing were the first to participate in the experiment in order to reduce the possibility that participants in this condition would be aware of the need to estimate time.

**Table 1:** Number of males and females in each condition.

	Gender		
Condition	Male	Female	Total
	Video game		
Prospective	27	32	59
Retrospective	37	42	79
	Reading		
Prospective	13	15	28
Retrospective	17	16	33

**Table 2:** Mean age (standard deviation) of participants in each condition.

	Paradigm	
Condition	Prospective	Retrospective
Video game	9.90 (1.37)	9.57 (.90)
Reading	8.86 (.80)	8.70 (.53)

Participants were presented with a brief description of the basic functioning of “The Sims” game. At the start signal, the children turned on their computer screens, chose between the two proposed families and began playing. At the same time, an experimenter started a chronometer and let children play for 14 minutes. In the prospective condition, the children were told just before they turned on the screen that they would have to estimate their playtime after the activity and that they should pay attention to time. At the end of the activity, the children first filled out the computerized questionnaire on time estimation. Then, an experimenter read them the questions from the other questionnaires one at a time. Essentially the same procedure was used with the readers. Just before the magazine was distributed to each child and the reading signal was given, the children were presented with a brief description of the magazine.

## Results

The results of the study are presented in three sections. The first section reports control analyses related to the age of the participants. The second one reports analyses of time estimations involving comparisons of paradigms (prospective vs. retrospective), tasks (video game vs. reading) and genders (female vs. male). The last section reports results related the tasks' appreciation and the perceived level of competency.

Before presenting the results, it should be mentioned that three time estimate dependent variables were used in analysis: (a) the estimated-to-target duration ratio, (b) the absolute standardized error and (c) a Weber Fraction like index. All three variables were used to assess the impact of the two independent variables (i.e. time estimation paradigm and task) on time estimates. These three variables will be further described below.

First, the estimated-to-target duration ratio (RATIO) was used to verify whether the direction of time estimates differed as a function of paradigm, task or gender. Thus, the RATIO was calculated by dividing the estimated duration (ED) by the target duration (TD, 14 minutes): RATIO = ED/TD. RATIO higher than one indicates that time is overestimated compared to real time.

Secondly, the absolute standardized error (ASE) was used to verify whether the amplitudes of the deviations of time estimates from real time differed across conditions. This statistic is an important measure of time estimation, for directional variables (like the RATIO) might miss timing performance differences across conditions [4]. The ASE was calculated by putting in absolute value the difference between the estimated duration (ED) and the target duration (TD), divided by the target duration (14 minutes): ASE = |(ED-TD)/TD|. Greater ASE indicates that time estimates were farther from the target duration, i.e. less accurate.

Thirdly, a WF-like index was used to determine whether the variability of time estimates differed across conditions. The WF was derived from the difference between the maximum (MAX) and minimum (MIN) time estimates (an estimate of variability), divided by the target duration (14 minutes): WF = (MAX-MIN)/TD. Higher WF indicates higher variability in the time estimates.

### Age

First of all, an analysis revealed that the age of participants did not differ as a function of gender. Indeed, female (*M* = 9.48, *SD* = 1.17) were not significantly older than male (*M* = 9.36, *SD* = 1.06), *t* (197) = −.722, *p*>.05.

Secondly, a series of Pearson correlations were executed to verify the presence of relationships between the age of participants and the three dependent variables (i.e. RATIO, ASE and WF). The results revealed that age was significantly related to the RATIO (*r* = .28, *p*<.001, *n* = 196), but not to the ASE (*r* = .04, *p*>.05, *n* = 196) or to the WF (*r* = .08, *p*>.05, *n* = 196). This positive correlation means that the older the participants are, the longer their time estimates are. Because of this result, it was decided to include the age as a covariate in the forthcoming ANOVA on the RATIO.

### Task, paradigm and gender comparisons

**Figure 1:**
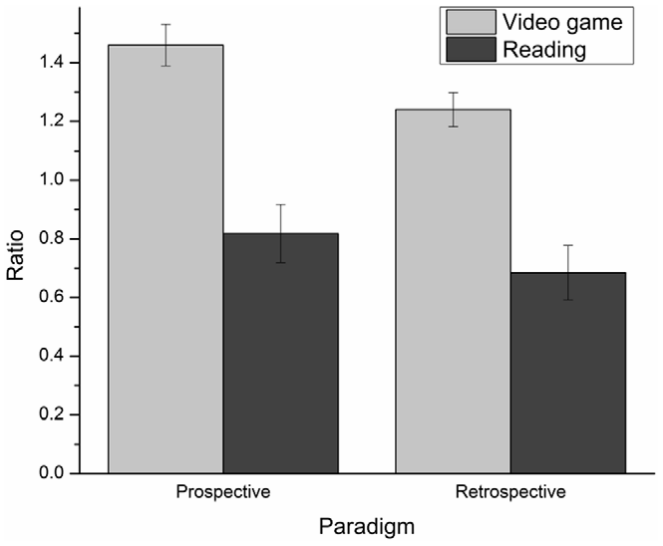
Ratio's mean and standard error for each task and for each paradigm.

Figure 1 presents RATIO's mean and standard error in each experimental condition. An ANCOVA conducted on the RATIO, with the age of participants included as a covariate, revealed a paradigm main effect: prospective RATIOs were larger than retrospective ones, *F*(1,187) = 4.81, *p*<.05, ŋ_p_^2^ = .03. Also, the ANCOVA revealed a task main effect: RATIOs in the gaming condition were larger than those in the reading condition, *F*(1,187) = 47.93, *p*<.001, ŋ_p_^2^ = .20. In addition, the age main effect was not significant, *F*(1,187) = 1.28, *p*>.05, ŋ_p_^2^ = .01. Finally, the gender main effect, *F*(1, 187) = 1.16, *p*>.05, ŋ_p_^2^ = .01, and the interaction effects were not significant.

**Figure 2:**
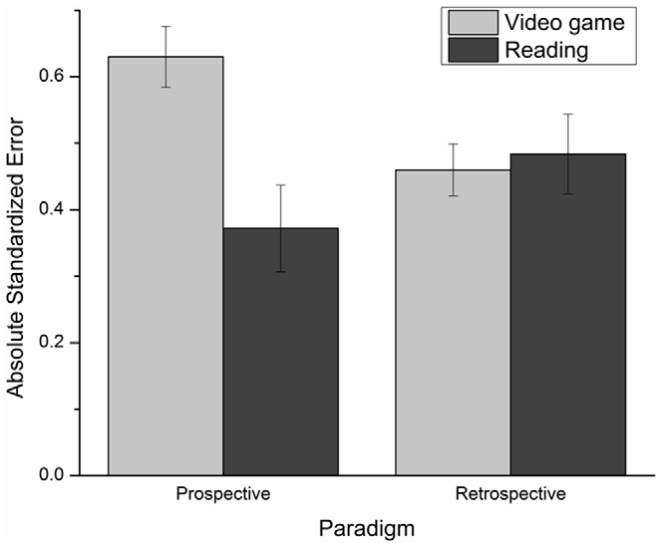
Absolute standardized error's mean and standard error for each task and for each paradigm.

Figure 2 presents ASE's mean and standard error in each experimental condition. The ANOVA conducted on the ASE revealed no paradigm main effect, *F*(1, 188) = .26, *p*>.05, ŋ_p_^2^ = .001, and no gender main effect, *F*(1, 188) = .19, *p*>.05, ŋ_p_^2^ = .001, but the task main effect was significant: ASEs were larger in the video game condition than in the reading task, *F*(1, 188) = 4.99, *p*<.05, ŋ_p_^2^ = .03. Finally, there was a paradigm×task interaction, *F*(1, 188) = 6.88, *p*<.05, ŋ_p_^2^ = .04. Subsequent t-test analyses revealed that the paradigm effect was only significant in the video game task: ASEs were larger in the prospective than in the retrospective condition, *t* (103.775) = 2.47, *p*<.05. It is noteworthy to mention that for this last comparison, degrees of freedom have been adjusted for unequal variances between groups.

**Figure 3:**
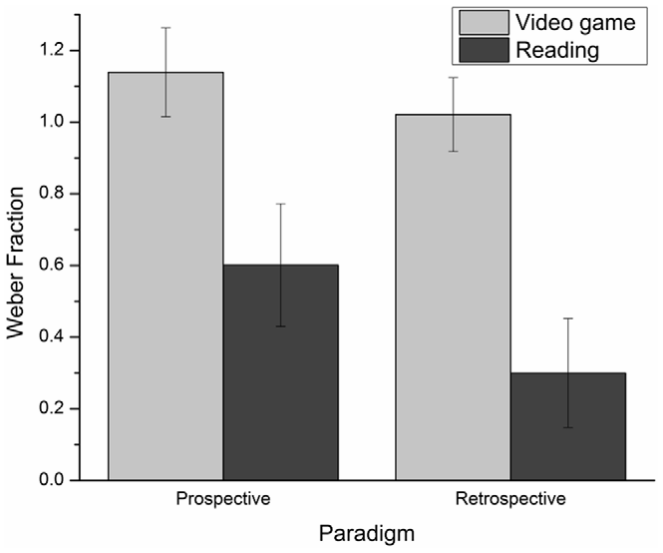
Weber Fraction's mean and standard error for each task and for each paradigm.

Figure 3 presents the WF's mean and standard error in each experimental condition. It is important to underline that some children have not estimated the likely minimum and maximum durations of the tasks. Thus, it was impossible to calculate a WF for these participants (*n* = 18). The ANOVA conducted on the WF revealed no paradigm main effect, *F*(1, 173) = 0.16, *p*>.05, ŋ_p_^2^ = .001. However, the task main effect, *F*(1, 173) = 6.80, *p*<.05, ŋ_p_^2^ = .04, and the gender main effect, *F*(1, 173) = 4.98, *p*<.05, ŋ_p_^2^ = .03, were significant: WFs were larger in the video game than in the reading task, and were larger for males than for females. Finally, interactions among factors were not significant.

### Tasks' appreciation and perceived level of competency

An ANOVA conducted on the task's appreciation revealed no main or interaction effect involving paradigm or task. The only significant effect was on the gender: girls (*M* = 5.49, *SD* = 1.33) reported higher level of enjoyment for the tasks than boys (*M* = 4.96, *SD* = 1.68), *F*(1, 191) = 8.815, *p*<.05, ŋ_p_^2^ = .04. Pearson correlations revealed that the task's appreciation was not related to the RATIO value (*r* = −.051, *p*>.05, *n* = 196) or to the ASE value (*r* = −.013, *p*>.05, *n* = 196), but was negatively and significantly related to WF (*r* = −.157, *p*<.05, *n* = 181).

In addition, given that (a) there was a significant effect of gender on the task appreciation level, (b) the task's appreciation level was significantly related to the WF and (c) there was a main effect of gender on the WF, it is important to verify if the task's appreciation mediates the gender effect on the WF. Pearson correlations revealed that the task's appreciation was not significantly related to the WF when these correlations were made independently for females (*r* = −.070, *p*>.05, *n* = 90) and for males (*r* = −.182, *p*>.05, *n* = 84). Therefore, it is reasonable to assume that the task's appreciation did not significantly mediate the influence of the gender on the WF.

An ANOVA conducted on the perceived level of competency revealed a task main effect: participant's level of competency was higher in the reading condition (*M* = 3.90, *SD* = .35) than in the video game condition (*M* = 2.38, *SD* = .71), *F*(1, 191) = 242.83, *p*<.001, ŋ_p_^2^ = .56. No other effect was significant. Pearson correlations revealed that the perceived level of competency of the task was negatively and significantly related to the RATIO value (*r* = −.373, *p*<.001, *n* = 196), but not to the ASE (*r* = −.036, *p*>.05, *n* = 196) or WF value (*r* = −.142, *p*>.05, *n* = 181).

In addition, given that (a) there was a significant task effect on the level of competency, (b) the level of competency was significantly related to the RATIO and (c) there was a main effect of task on the RATIO, it is important to verify if the level of competency mediates the effect of the task on the RATIO. Pearson correlations revealed that the perceived level of competency was not significantly related to the RATIO when these correlations were made independently for the video game condition (*r* = .009, *p*>.05, *n* = 135) and for the reading condition (*r* = −.105, *p*>.05, *n* = 61). Therefore, it is reasonable to assume that the level of competency did not significantly mediate the influence of the task on the RATIO.

## Discussion

The main goals of the present study were (a) to compare prospective and retrospective time estimates in two ecologically valid tasks with a children sample and (b) to verify if playing video games induces an underestimation of time compared to another pleasant task. The present discussion will address each of these goals. Moreover, a developmental perspective on time estimation of long lasting ecological tasks will be presented. Finally, some of the present study limitations will be discussed.

### Paradigm comparisons within ecological tasks

Consistent with our hypothesis regarding paradigms comparison, the experiment revealed that regardless of the tasks (i.e. video gaming or reading), prospective time estimates were longer (higher RATIO) and not more or less variable (no significant differences on the WF) than retrospective ones. The overestimation of time in the prospective paradigm is consistent with the normal trend seen in the literature: prospective time estimates are longer than retrospective ones [3]. As for variability, prospective time estimates are normally reported to be less variable [3], but this trend is mostly based on short durations. Still, the results of the present study are consistent with Tobin et al. [15]. Indeed, the authors reported that prospective time estimates of 35- and 58-min of video gaming were longer (ratios of about 1.2) than retrospective ones (ratios of about .95) and that variability of time estimates did not differ significantly as a function of paradigms.

Contrary to our hypothesis, the present results showed that prospective time estimates were less accurate (i.e. higher ASEs) than retrospective ones in the video game condition only. These results could partially be explained by the fact that the magnitude of time estimate errors was greater in the prospective video game condition. Indeed, as indicated in Figure 2, the ASE mean in this condition was much larger than those in the three other conditions. The possible explanation regarding the difference in the magnitude of time estimate errors will be addressed in the next section, as tasks' comparison results will be discussed.

### Tasks comparison

One other aim of this study was to verify if time would be underestimated in the video game conditions compared to the reading conditions. Surprisingly, this hypothesis was not confirmed as the video game task was overestimated. Indeed, results revealed that reading not only led to much smaller time estimates than video gaming, but also resulted in large underestimations of time in both prospective and retrospective reading conditions (see Figure 1). Moreover, time estimates in the video game conditions were less accurate (i.e. higher ASEs) and more variable (i.e. higher WF) than those of the reading conditions. Most adults know that there is nothing like a good book for killing time. This seems to apply to children as well; in fact, reading seems to work even better than playing a video game.

This finding in the prospective condition is particularly surprising when one considers that gaming should have been perceived more pleasant than reading and thus should have captured the attention of participants and detracted it from time. On the contrary, the level of appreciation of both tasks did not differ significantly. Still, the reading condition led to much lower time estimates and has seemed to detract attention from time more than the video game. Detracting attention from time is known to lead to underestimations of time [6].

On the other hand, our finding is somewhat consistent with Rau et al. [30], who reported that novice players overestimate a 60-min period of play. In fact, recent observations showed that gamers tested in prospective conditions in video game centers overestimated 12-, 35- and 58-min playing periods [15]. Also, Tobin and Grondin [22] observed an overestimation of an 8-min play, but underestimation of a 24-min period by teenagers playing a video game.

One element that could explain the results is the fact that, as discussed earlier, time estimates error amplitudes (i.e. ASEs) in the prospective video game condition are much higher than in the retrospective one or than in both reading conditions (i.e., prospective and retrospective conditions). The nature of the video game task could partially account for the observed results. In fact, since participants are aware that they are prone to lose track of time [29], they may try to adjust their estimates to compensate for this loss of temporal consciousness. Thus, this adjustment could explain why the ASE was larger for the video game task, particularly in the prospective condition.

Another hypothesis that could account for this time overestimation was proposed by Tobin et al. [15], who argued that video gamers might require an “adaptation period” to become fully immersed in a game. This hypothesis would apply especially when gamers start a new scenario (with new characters) as in the case of the game session (“The Sims”) used in this study. This adaptation period might be less pleasant and may thus induce the unexpected overestimation of time, with novice players being more likely to make overestimates (as in [30]). Two elements of results could partially support this hypothesis. First of all, participants of the reading condition reported higher competency levels compared to participants in the video game condition. Secondly, competency level was negatively correlated with RATIO scores, meaning higher was the competency level, lower were time estimates.

Finally, it is impossible to reject the hypothesis that the process of time estimation, be it prospective or retrospective, is special in the case of video games. This hypothesis could partially be supported by the fact that the video game task led to higher variability (i.e. higher WF) in time estimates, regardless of the paradigms (prospective vs. retrospective). In fact, as the duration was the same for both tasks, the variability should have been constant across conditions, which was not the case. On a general basis, variability is studied in the context of multiple duration comparison studies (e.g. comparing the variability around estimates of 1, 2, 4, or 8 sec). In those cases, when variability is not proportionally constant across duration conditions, it is sometime proposed that different processes are involved in the treatment of the durations for which variability differs from other durations [31]. Even though this hypothesis needs to be taken cautiously, it may also apply to task differences, thus in the case of video games.

### Developmental perspective

The results of the present study allow to tentatively identify developmental similarities in the context of time estimation of long lasting ecological tasks. It seems that for both children (this study) and adults [15], prospective time estimates are longer than retrospective ones. This observation enhanced the fact that, regardless of the age and the task used, attention plays an important role in timing processes [6].

Also of interest is the fact that the prospective time estimates were not more or less variables than retrospective ones. This result is also consistent with the data obtained by Tobin et al. [15] with a young adult sample. Once again, regardless of the age group or of the fact that different video games were used in both studies, the variability around time estimates of long lasting ecological tasks does not seem to vary across paradigms.

In addition, children (the present study), adolescents [22] and young adults [15] seem to overestimate short periods of video game play time (14, 8 and 12 minutes respectively). This trend could indicate that, regardless of a person's age, the video game played (in fact, all three studies used different video games) or the time estimation paradigm, the experience of time underestimation while playing video games does not seem to occur within the first 15 minutes of a play period. On the contrary, time seems to be overestimated during these first few minutes of play. Moreover, the RATIO values in the three experiments are quite similar. Indeed, for all three studies, RATIO means range between 1.3 and 1.6, regardless of the age group, the video game played or the paradigm used. This observation could indicate that when playing video games for a short amount of time, children, adolescents and adults verbally overestimate time in a similar way. Surprisingly, this trend is not consistent with previous reports in the literature. Indeed, some authors reported that children tend to make larger verbal estimates than adolescents and adults [32]. Moreover, not only children verbal estimates are similar to those of adults, but the amplitude of the time estimate errors (i.e. the ASE) are also similar. In fact, a comparison of the ASEs reported in the present study and in Tobin et al. [15] allows to propose that both age groups tend to have similar error amplitudes when judging the duration of short video game playing times.

In brief, the trends underlined in this developmental comparison seem to indicate that in the context of short video games, adolescents, young adults and children of 8–9 years old verbally estimate playing time in a similar manner. Contrary to what has been previously reported [32], this would mean that time estimation mechanisms implied in long lasting ecological tasks are already in place at the end of childhood, at least when the verbal estimation method is used. Nevertheless, although interesting, these developmental comparisons need to be confirmed by other studies comparing directly prospective and retrospective time estimates of children, adolescents and adults.

### Limitations of the present study

The present study presents some limitations. One of those is the fact that only one target duration was used to test the different hypotheses. This limitation is relevant when one considers that time estimation processes may differ as a function of durations [33]. Also, Tobin et al. [15] reported evidence that time estimates differ as a function of used durations. Indeed, the authors showed that young adults proportionally overestimated a 12-min video game play period compared to a 35- and a 58-min game play periods. Thus, the present results may not apply to other durations.

Another limitation regards the difficulty to relate time estimation results to any processes involved in timing (e.g. attention, memory, emotions and level of immersion in the game). Indeed, the use of ecological, and therefore uncontrolled tasks, makes the measurement of variables normally reported to influence time estimations a difficult undertaking. For example, one could raise the argument that, compared to the reading task, the video game task raised participants' level of arousal, which in turn could have accelerated the internal clock, thus resulting in the overestimation of time in the video game conditions. That hypothesis would be supported by studies reporting that higher levels of arousal are associated with longer estimation of time compared to lower levels of arousal [5], [34], [35].

Another hypothesis that could explain the results is that the reading task was more cognitively demanding than the video game task, at least for this sample. Once again, as more cognitive resources (e.g. attention) are invested in the task at hand (e.g. reading), less are left for the time estimation task. Consequently, participants in the reading condition underestimated time compared to the video game condition. Detracting attention from time is known to lead to underestimations of time [6]. However, this finding is supported in the time perception literature mainly by studies concerned with brief intervals (of less than a minute and often in the range of a few seconds). Interestingly, this cognitive load hypothesis could explain why the video game condition in Tobin and Grondin's [22] study was underestimated compared to reading and that the opposite was observed in the present study. Even though both studies used a video game, it is possible that the cognitive demands of the “Tetris” game used in Tobin and Grondin's [22] differ from “The Sims” used in the present study. Perhaps these differences, which were not objectively measured in both studies, could explain why the video game periods were underestimated in the former study and not in the present one.

Even though it needs to be taken cautiously, this last hypothesis shows the need for future research on long time estimations to emphasize the use of ecological tasks and the measure of variables like the cognitive load or the level of arousal. This measurement could be achieved with validated questionnaires or physiological measures. These approaches should enhance our understanding of time estimate processes within the context of long ecological tasks.

### Conclusion

Albeit the limitations discussed earlier, the present study is, to our best knowledge, the first to compare children's prospective and retrospective time estimates of ecological tasks. Thus, it could establish the basis for future research interested in developmental comparison of time estimation capabilities of ecological tasks. Moreover, the use of video gaming and reading as ecological tasks provided an opportunity to compare children's time estimates in two different activities. Also, the use of a video game opened the opportunity to explore relationships between video gaming profile and time estimate variables. In brief, the present study showed that: (a) children's prospective time estimates of two ecological tasks were longer than retrospective ones, but not more or less accurate or variable than retrospective ones; (b) the time estimates for the video game task were longer, less accurate and more variable than those of the reading task; and (c) compared to adolescents and adults, children seems to have the same abilities to verbally estimate the duration of short video game play periods. Finally, the limitations discussed enhanced the importance of extending the range of durations investigated and of documenting the differences in the cognitive processes involved in the ecological tasks used in time estimation studies.
